# The Impact of Nontuberculous Mycobacteria Species on Mortality in Patients With Nontuberculous Mycobacterial Lung Disease

**DOI:** 10.3389/fmicb.2022.909274

**Published:** 2022-07-07

**Authors:** Ping-Huai Wang, Sheng-Wei Pan, Su-Mei Wang, Chin-Chung Shu, Chin-Hao Chang

**Affiliations:** ^1^Division of Thoracic Medicine, Far Eastern Memorial Hospital, New Taipei City, Taiwan; ^2^Department of Nursing, Asia Eastern University of Science and Technology, New Taipei City, Taiwan; ^3^Department of Chest Medicine, Taipei Veterans General Hospital, Taipei, Taiwan; ^4^School of Medicine, National Yang Ming Chiao Tung University, Taipei, Taiwan; ^5^Department of Medical Research, National Taiwan University Hospital, Taipei, Taiwan; ^6^Department of Internal Medicine, National Taiwan University Hospital, Taipei, Taiwan; ^7^College of Medicine, National Taiwan University, Taipei, Taiwan

**Keywords:** chronic airway disease, species, *Mycobacterium kansasii*, mortality, nontuberculous mycobacteria, non-cancer, lung disease

## Abstract

Patients with nontuberculous mycobacterial lung disease (NTM-LD) have increased mortality. The impact of NTM species on the risk of mortality remains unclear, especially that of death by non-cancer causes. We conducted a retrospective cohort study from 2006 to 2018 in a tertiary-care hospital in Taiwan. We enrolled patients who fulfilled the microbiological diagnostic criteria of NTM-LD. The mortality causes within 8 years after diagnosis were identified, and the Cox proportional hazard regression was performed for risk factors of mortality. A total of 1,652 subjects with NTM-LD were included. Among them, 723 (43.8%) were infected by *Mycobacterium avium* complex (MAC), 408 (24.7%) by *M. abscessus* complex (MABC), 120 (7.3%) by *Mycobacterium kansasii* (MK), 304 (18.4%) by other rapid-growing mycobacteria (RGM), and 97 (5.9%) by other slow-growing mycobacteria (SGM) groups. The 8-year all-cause mortality was 45.2% for all and the highest in the MK-LD group (59.2%), followed by the MABC-LD and MAC-LD groups. The adjusted hazard ratios were 2.20 (95% confidence interval: 1.40–3.46) in the MK-LD, 1.85 (1.54–2.22) in the MABC-LD, and 1.65 (1.12–2.41) in the MAC-LD groups for all-cause mortality, compared with the SGM group. Kaplan–Meier survival curves showed that all-cause mortality, non-cancer mortality, and mortality due to chronic airway diseases were significantly correlated with NTM species (log-rank *p* = 0.0031, < 0.001, and 0.001, respectively). High 8-year mortality rates were found in patients with NTM-LDs according to different NTM species. Notably, the difference was significant in non-cancer mortality causes, especially in chronic airway diseases.

## Introduction

Nontuberculous mycobacteria (NTM) isolates in respiratory specimens were previously considered harmless colonizers of the airway, and NTM-lung disease (NTM-LD) was treated as an opportunistic infection in immunocompromised patients (Horsburgh, 1996). However, some studies have demonstrated that NTM-LD is not only present in patients with immune deficits but also gradually progresses and causes significant mortalities in patients with comorbidities (Winthrop et al., 2010). Importantly, although the mortality related to NTM-LD is not as high as that of *Mycobacterium tuberculosis* (Mtb), the incidence of NTM-LD continues to increase, while Mtb infection has decreased gradually since the implementation of public health policies in many regions (Winthrop et al., 2010). Notably, the British Thoracic Society (BTS) and the American Thoracic Society (ATS)/Infectious Disease Society of America (IDSA), respectively, released guidelines on NTM-LD diagnosis and treatment in 2017 and 2020 to improve the outcomes of patients with NTM-LD (Haworth et al., 2017; Daley et al., 2020).

Considering the prognosis of patients with NTM-LD, the 5-year all-cause mortality rate was around 13–45% (Novosad et al., 2017; Lee et al., 2021; Mourad et al., 2021), and the risk of all-cause mortality in patients with NTM-LD was two times that of controls (Marras et al., 2018). Disease severity and several host-related factors, including low body mass index (BMI), high inflammatory markers, cavitation, and extensive lung involvement, are poor prognostic factors in patients with NTM-LD (Ito et al., 2012; Pan et al., 2017; Kim et al., 2021). Apart from the severity of NTM-LD itself, certain comorbidities are correlated with a worse prognosis in patients with NTM-LD (Jhun et al., 2020; Lee et al., 2021; Mourad et al., 2021).

The NTM species also might be a prognostic factor. A study in Canada reported various hazard ratios (HR) of all-cause mortality rates among different NTM species to the general population but provided no details on causes of death (Marras et al., 2017). Some studies have reported the specific mortality causes of certain NTM-LDs, but those studies lacked comparisons of the relationship between mortality and NTM species (Hwang et al., 2017; Liu et al., 2019). A population-based study in Korea reported the distribution of mortality causes in NTM-LD mortality in a 15-year follow-up study, but they focused on only *Mycobacterium avium* complex (MAC) and *M. abscessus* complex (MABC) (Lee et al., 2021). In a long-term follow-up, information about causes of death could provide a further understanding of the disease course. The information on the relationship between NTM species and mortality will need to be developed through the further comprehensive investigation to help clinicians prioritize patients at risk of worse prognosis for treatment and follow-up. Therefore, we conducted this large-scale cohort study to analyze the risk factors of NTM-LD mortality, focusing on the impact of NTM species on different mortality causes.

## Materials and Methods

### Study Design and Population

We conducted this retrospective cohort study from January 2006 to June 2018 at a tertiary-care hospital in Taiwan. We reviewed subjects who had been examined by sputum mycobacterial culture by drawing patient data from the hospital’s Integrated Medical Database. We included subjects who fulfilled the microbiological diagnostic criteria of NTM-LD according to the ATS/IDSA diagnosis guideline (Griffith et al., 2007) (Supplementary Appendix 1). We excluded patients infected by HIV (+) and those who died within the first month of the diagnosis of NTM-LD.

The hospital’s Research Ethics Committee approved this study (No: 201704001RINB and 202007084RINA). All the personal data were delinked, and informed consent was waived due to the retrospective nature of the study.

### Definition, Groups, and Follow-up

Microbiologically, sputa were examined using Ziehl–Neelsen stain (acid-fast stain, AFS). The results of sputum smears were reported semiquantitatively from trace to four positive according to the diagnostic standard recommendations of American Thoracic Society and the Centers for Disease Control (2000). The grades of AFS between a trace to 2+ were defined as weak positive, and those of 3+ or 4+ were defined as a strong positive. We categorized NTM species as MABC, MAC, *Mycobacterium kansasii* (MK), other rapidly growing mycobacteria (RGM), and other slowly growing mycobacteria (SGM) groups (Supplementary Appendix 2). The presence of NTM treatment was defined as a treatment regimen of more than 8 weeks with ≥ 2 effective antibiotics recommended by the guidelines (Daley et al., 2020). The index date was defined as the first date of NTM isolates. The follow-up time frame was from the index date to the date of the last hospital visit or 8 years. Cavitation was defined by computed tomography (CT). In the aspect of comorbidities, chronic kidney disease was considered if the estimated glomerular filtration rate (eGFR) was ≤ 45 ml/min/1.73 m^2^. Other comorbidities were coded by a diagnosis of medical records (Supplementary Appendix 3).

### Outcomes

The primary outcome was all-cause mortality. The date of death and mortality causes were obtained from the death statistics database of the Ministry of Health and Welfare, Taiwan (Hsiao et al., 2015). We then analyzed its risk factors, focusing on the impact of NTM species. The secondary outcome was death due to other causes. Specifically, the mortality causes were categorized into seven classes, including cancer, chronic airway diseases, pneumonia, cardiovascular diseases, cerebrovascular disease, diabetes mellitus (DM), and others (Supplementary Appendix 4). The mortality related to chronic airway disease was the summation of mortality due to chronic obstructive pulmonary disease (COPD), asthma, pneumoconiosis, bronchiectasis, and idiopathic pulmonary fibrosis (IPF) (Lee et al., 2021).

### Statistical Analysis

All statistical analyses were conducted in SAS 9.4 (Cary, NC, United States). Categorical and continuous variables were compared using the Chi-squared and Student’s *t*-tests, respectively. ANOVA was used for the comparison of multiple groups. The mortality rates were estimated using the Kaplan–Meier (KM) method. Univariable hazard ratios (HRs) were calculated by Cox proportional hazard regression. In considering multiple comparisons and statistical power loss, factors in univariable Cox proportional hazard regression with *p*-values of < 0.0033 (0.05/15 variables) were included in the multivariable analysis, according to Bonferroni correction. KM survival curves and log-rank tests were used for the analysis of survival curves. The KM survival curves between two NTM species were compared using a pairwise log-rank test. Statistical significance was set at *p* < 0.05.

## Results

### Subjects’ Enrollment

A total of 62,956 subjects with positive mycobacterial cultures were reviewed. Among them, 2,042 subjects fulfilled the microbiological diagnostic criteria (Supplementary Figure 1). We excluded 100 subjects because they died within 1 month of NTM-LD diagnosis. In addition, 231 subjects were excluded because they were diagnosed as having concomitant PTB. We also excluded 59 subjects who were referred only for sputum mycobacteria cultures. Thus, 1,652 subjects were finally enrolled, including 723 (43.8%) patients infected with MAC, 408 (24.7%) by MABC, 120 (7.3%) by MK, 304 (18.4%) by RGM, and 97 (5.9%) by SGM.

### Demographics

The median follow-up period was 3.61 (lower quartile to upper quartile: 1.35–6.45) years (Table 1). The 1, 5, and 8-year mortality rates were 15.7, 35.8, and 45.2%, respectively. A total of 591 subjects died within the follow-up period (mortality group), and the remaining 1,061 survived (survival group). The mortality group was older, had a BMI of < 18.5 kg/m^2^, and comprised more males than the survival group. Regarding comorbidities, more subjects had CKD, COPD, pneumoconiosis, IPF, cancer, cirrhosis, congestive heart failure (CHF), cerebrovascular accident (CVA), and DM in the mortality group than in the survival group. In contrast, fewer subjects had bronchiectasis and borderline higher cavitation in the mortality group than in the survival group. The grades of AFS and the proportion of subjects with anti-NTM treatment were not significantly different between the two groups. One-way ANOVA showed the NTM species to be significantly different between the mortality and survival groups (*p* = 0.036).

**TABLE 1:** Characteristics of all subjects and subgroups with survival and mortality outcomes.

Characteristics	All *n* = 1652	Survival *n* = 1061	Mortality *n* = 591	*P*-value
**Age**	66.9 ± 14.4	63.7 ± 13.3	72.5 ± 14.6	< 0.001
**Male**	859 (52.0)	454 (42.8)	405 (68.5)	< 0.001
BMI*, kg/m^2^	21.1 ± 4.0	21.9 ± 3.8	20.7 ± 4.1	< 0.001
**BMI < 18.5** kg/m^2^	224 (24.0)	105 (18.8)	119 (31.9)	< 0.001
Smoking*(ever/non-smoker)	279/1127 (19.8/80.2)	141/795 (15.1/84.9)	138/332 (29.3/70.6)	< 0.001
**AFS, *n* = 1622**				0.57
Negative	1377 (84.9)	892 (85.4)	485 (83.9)	
Positive (weak)	183 (11.3)	116 (11.1)	67 (11.6)	
Positive (strong)	62 (3.8)	36 (3.4)	26 (4.5)	
**Mycobacterial species^#^**				0.036
MAB	408 (24.7)	254 (62.3)	154 (37.7)	
MAC	723 (43.8)	468 (64.7)	255 (35.3)	
MK	120 (7.3)	64 (53.3)	56 (46.7)	
RGM	304 (18.4)	208 (68.4)	96 (31.6)	
SGM	97 (5.9)	67 (69.1)	30 (30.9)	
**Cavity lesion**	115 (7.0)	65 (6.1)	50 (8.5)	0.074
**Comorbidities**				
CKD	120 (7.3)	28 (2.6)	92 (15.6)	< 0.001
Chronic airway disease	629 (38.1)	432 (40.7)	197 (33.3)	0.003
COPD	229 (13.9)	112 (10.6)	117 (19.8)	< 0.0001
Asthma	105 (6.4)	72 (6.8)	33 (5.6)	0.34
Bronchiectasis	355 (21.5)	296 (27.9)	59 (10.0)	< 0.001
Pneumoconiosis	16 (1.0)	6 (0.6)	10 (1.7)	0.025
IPF	43 (2.6)	20 (1.9)	23 (3.9)	0.014
Cancer	366 (22.2)	150 (14.1)	216 (36.6)	< 0.001
Cirrhosis	29 (1.8)	12 (1.1)	17 (2.9)	0.010
ESRD	49 (2.97)	12 (1.13)	37 (6.26)	< 0.001
CHF	81 (4.9)	20 (1.9)	61 (10.3)	< 0.001
Stroke	37 (2.2)	12 (1.1)	25 (4.2)	< 0.001
DM	198 (12.0)	94 (8.9)	104 (17.6)	< 0.001
Autoimmune disease	64 (3.9)	44 (4.2)	20 (3.4)	0.44
Transplant	34 (2.1)	18 (1.7)	16 (2.7)	0.17
**NTM treatment**	211 (12.8)	136 (12.8)	75 (12.7)	0.94
**Duration of treatment, days**	294.5 ± 215.2	300.4 ± 185.2	283.7 ± 262.1	0.63
**Follow-up duration, years^†^**	3.61 (1.35–6.45)	5.30 (2.68–8.0)	1.31 (0.40–3.06)	< 0.001

*Data are presented as mean ± standard deviation or number (percentage).*

*^†^Data are presented as median (lower quartile–upper quartile).*

*There were 932 patients with BMI data and 1,406 with smoking coding.

*^#^The denominators of survival and mortality rate are the case numbers of specific nontuberculous mycobacteria. BMI, body mass index; AFS, acid fast staining; MABC, Mycobacterium abscessus complex; MAC, Mycobacterium avium complex; MK, Mycobacterium kansasii; RGM, rapid-growing mycobacteria; SGM, slow-growing mycobacteria; NTM, nontuberculous mycobacteria; CKD, chronic kidney disease; COPD, chronic obstructive pulmonary disease; IPF, idiopathic pulmonary fibrosis; ESRD, end-stage renal disease; CHF, heart failure; DM, diabetes mellitus. p, survival vs. mortality group.*

### Risk Factors of Nontuberculous Mycobacterial Lung Disease Mortality

Univariable Cox proportional hazard regression analysis reported that male, age ≥ 65 years, ever-smoking, and pulmonary cavitation were the risk factors for NTM-LD mortality (Table 2). Comorbidities, including CKD, COPD, pneumoconiosis, IPF, cancer, cirrhosis, CHF, CVA, and DM, also had significantly greater impacts on NTM-LD mortality. Patients with bronchiectasis seemed to have less mortality [crude hazard ratio (HR): 0.37, 95% CI: 0.28–0.49]. Compared to those with negative sputum AFS, neither a weak nor a strong sputum AFS score was a significant risk factor for NTM-LD mortality. Notably, NTM species were correlated with the risk of NTM-LD mortality. Compared to SGM species, the crude HRs for mortality in the MABC, MAC, and MK groups were 1.52 (95% CI: 1.03–2.25), 1.36 (0.93–1.98), and 1.97 (1.27–3.07), respectively.

**TABLE 2:** Univariable and multivariable Cox proportional hazard regression on the risk factors of NTM-LD mortality.

Variables	Crude HR (95% CI)	*P*-value	Adjusted HR* (95% CI)	*P*-value
Age≥65 years	2.30 (1.93–2.76)	< 0.001	1.80 (1.49–2.18)	< 0.001
Sex, Male	2.32 (1.95–2.76)	< 0.001	1.85 (1.54–2.22)	< 0.001
BMI				
BMI < 18.5 kg/m^2^	1.79 (1.43–2.22)	< 0.001		
BMI ≥ 18.5 kg/m^2^	Reference			
Smoke				
Ever-smoker	2.09 (1.71–2.55)	< 0.001		
Non-smoker	Reference			
AFS grading		0.079		
Negative	Reference			
Positive (weak)	1.23 (0.95–1.58)	0.12		
Positive (strong)	1.42 (0.96–2.11)	0.080		
Mycobacterial species				
MABC	1.52 (1.03–2.25)	0.035	1.85 (1.24–2.74)	0.0024
MAC	1.36 (0.93–1.98)	0.11	1.65 (1.12–2.41)	0.010
MK	1.97 (1.27–3.07)	0.0027	2.20 (1.40–3.46)	< 0.001
RGM	1.13 (0.75–1.70)	0.57	1.20 (0.79–1.81)	0.39
SGM	Reference		Reference	
Cavity	1.36 (1.01–1.81)	0.040		
Old TB	1.14 (0.83–1.57)	0.40		
CKD	3.28 (2.62–4.10)	< 0.001	2.07 (1.56–2.74)	< 0.001
Chronic airway disease				
COPD	1.74 (1.42–2.13)	< 0.001	0.93 (0.75–1.16)	0.52
Asthma	0.85 (0.60–1.21)	0.38		
Bronchiectasis	0.37 (0.28–0.49)	< 0.001	0.56 (0.42–0.73)	< 0.001
Pneumoconiosis	2.18 (1.17–4.07)	0.015		
IPF	1.84 (1.21–2.79)	0.0043		
Cancer	2.85 (2.41–3.37)	< 0.001	2.55 (2.15–3.03)	< 0.001
Cirrhosis	2.36 (1.45–3.82)	< 0.001	1.35 (0.82–2.22)	0.23
ESRD	3.00 (2.15–4.19)	< 0.001	1.25 (0.84–1.88)	0.27
CHF	3.66 (2.81–4.78)	< 0.001	2.31 (1.74–3.08)	< 0.001
Stroke	2.18 (1.46–3.25)	< 0.001	1.08 (0.72–1.64)	0.70
DM	1.86 (1.50–2.29)	< 0.001	1.28 (1.03–1.59)	0.028
Autoimmune	0.95 (0.61–1.48)	0.23		
Transplant	1.47 (0.90–2.42)	0.13		
NTM treatment	0.93 (0.73–1.18)	0.54		

*Corrected by Bonferroni correction. BMI, body mass index; AFS, acid-fast staining; HR, hazard ratio; MABC, Mycobacterium abscessus complex; MAC, Mycobacterium avium complex; MK, Mycobacterium kansasii; RGM, rapid-growing mycobacteria; SGM, slow-growing mycobacteria; NTM, nontuberculous mycobacteria; CKD, chronic kidney disease; COPD, chronic obstructive pulmonary disease; IPF, idiopathic pulmonary fibrosis; ESRD, end-stage renal disease; CHF, heart failure; DM: diabetes mellitus.

The results of multivariable Cox proportional regression with Bonferroni correction are shown in Table 2. Smoking status and BMI were used for sensitivity analysis but not for multivariable analysis because of some missing data. Male, age ≥ 65 years, NTM species [MABC (adjusted HR: 1.85, 95% CI: 1.24–2.74), MAC (1.65, 1.12–2.41), and MK (2.20, 1.40–3.46) compared to SGM], and the comorbidities of CKD, cancer, CHF, and DM were independent risk factors for mortality. Among the significant comorbidities, patients with bronchiectasis had less mortality (adjusted HR: 0.56, 0.42–0.73).

**FIGURE 1:**
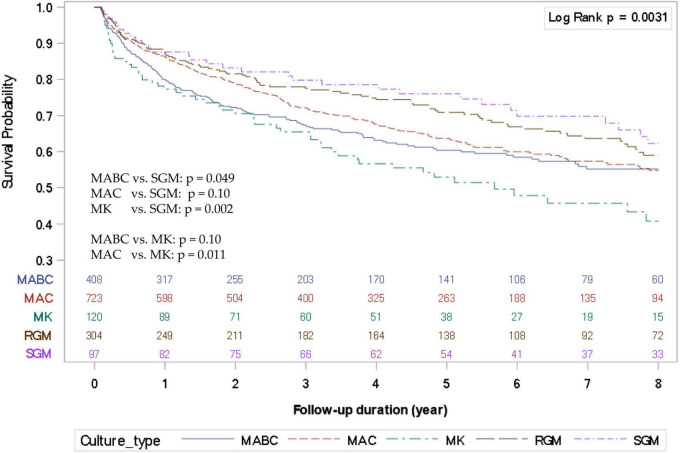
Kaplan–Meier survival curves of nontuberculous mycobacterial lung disease (NTM-LD) according to different NTM species. MABC, *Mycobacterium abscessus* complex; MAC, *Mycobacterium avium* complex; MK, *Mycobacterium kansasii*; SGM, slow-growing mycobacteria.

The KM survival curves among different NTM species were significantly different (log-rank *p* = 0.0031) (Figure 1). Compared to the SGM group, the survival rates of MABC-LD and MK-LD were significantly lower (Supplementary Table 1), but only a borderline trend was evident in MAC-LD (*p* = 0.10). However, the KM survival curve of MK-LD was not significantly different from that of MABC-LD.

**FIGURE 2:**
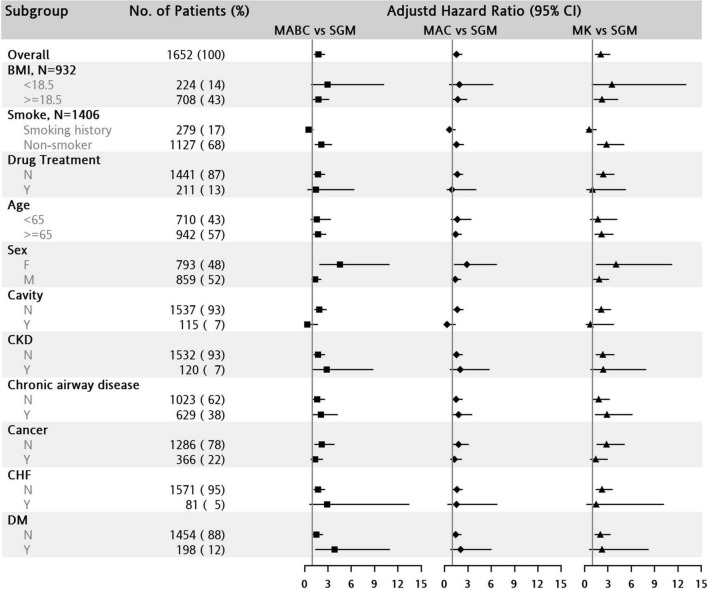
Subgroup analysis of hazard ratio for all-cause mortality in patients with nontuberculous mycobacterial lung disease (NTM-LD). The reference group is the slow-growing mycobacteria (SGM) group. BMI, body mass index; CHF, congestive heart failure; CKD, chronic kidney disease; DM, diabetes mellitus; MABC, *Mycobacterium abscessus* complex; MAC, *Mycobacterium avium* complex; MK, *Mycobacterium kansasii.*

The subgroup sensitivity analysis of adjusted HR for 8-year mortality in the MABC, MAC, and MK groups was compared with SGM (Figure 2). Regardless of BMI < 18.5, old age, sex, and comorbidities, the MABC, MAC, and MK groups had significantly higher risks of mortality than the SGM group.

### Characteristics of Patients With Nontuberculous Mycobacterial Lung Disease Caused by Different Nontuberculous Mycobacteria Species

Patients with MAC-LD were the oldest on average (68.6 ± 13.6 years), whereas subjects with MABC-LD were the youngest (64.4 ± 15.5 years, one-way ANOVA *p* < 0.001) (Table 3). Patients with MK-LD had male predominance, lower BMI, more ever smoking, radiographical cavity, underlying disease of COPD and pneumoconiosis diagnosis, as well as a higher rate of initiating NTM treatment (19.2%, *p* < 0.001). Patients with MABC-LD had female predominance, higher positive AFS, and more underlying diseases of both old TB and DM, whereas those with MAC-LD had lower BMI, more old TB, and bronchiectasis. More subjects in the RGM and SGM groups had cancer, asthma, and lower treatment rates. Specifically, for the different NTM groups, the 8-year mortality rates were highest in the MK group (59.2%), followed by the MAC group (45.2%) and the MABC group (44.8%).

**TABLE 3:** Characteristics of patients with NTM-LD stratified by NTM species.

Characteristics	MABC 408 (24.7)	MAC 723 (43.8)	MK 120 (7.3)	RGM 120 (7.3)	SGM 97 (5.9)	*P*-value
**Age**	64.4 ± 15.5	68.6 ± 13.6	66.1 ± 16.0	66.3 ± 14.3	66.9 ± 12.0	< 0.001
Age ≥ 65	200 (49.0)	444 (61.4)	68 (56.7)	175 (57.6)	55 (56.7)	0.003
**Male,** no (%)	209 (51.2)	349 (48.3)	85 (70.8)	166 (54.6)	50 (51.6)	< 0.001
**BMI**, kg/m^2^, *n* = 932	21.9 ± 4.1	20.7 ± 3.8	20.5 ± 3.6	22.5 ± 3.8	23.1 ± 4.3	< 0.001
BMI < 18.5 kg/m^2^	56 (22.9)	118 (29.1)	21 (28.0)	21 (13.7)	8 (15.1)	0.001
**Ever smoking,** no (%), *n* = 1406	73 (21)	105 (17)	38 (38)	51 (20)	12 (14)	< 0.001
**AFS**, no (%), *n* = 1622						< 0.001
Negative	320 (79.6)	596 (84.42)	97 (80.83)	273 (91.3)	91 (95.8)	
Positive (weak)	57 (14.2)	86 (12.2)	16 (13.3)	21 (7.0)	3 (3.2)	
Positive (strong)	25 (6.2)	24 (3.4)	7 (5.8)	5 (1.7)	1 (1.1)	
**Cavity,** no (%)	24 (5.9)	55 (7.6)	14 (11.7)	19 (6.3)	3 (3.1)	0.10
**Comorbidities,** no (%)						
Old TB	30 (7.4)	52 (7.2)	3 (2.5)	11 (3.6)	2 (2.1)	0.021
CKD	29 (7.1)	53 (7.3)	14 (11.7)	17 (5.6)	7 (7.2)	0.32
Chronic airway disease						
COPD	40 (9.8)	101 (14.0)	29 (24.2)	44 (14.5)	15 (15.5)	0.002
Asthma	21 (5.2)	41 (5.7)	5 (4.2)	29 (9.5)	9 (9.3)	0.058
Bronchiectasis	93 (22.8)	186 (25.7)	21 (17.5)	41 (13.5)	14 (14.4)	< 0.001
Pneumoconiosis	1 (0.3)	2 (0.3)	5 (4.2)	7 (2.3)	1 (1.0)	< 0.001*
IPF	11 (2.7)	18 (2.5)	4 (3.3)	7 (2.3)	3 (3.1)	0.97
Cancer	95 (23.3)	142 (19.6)	21 (17.5)	80 (26.3)	28 (28.9)	0.040
Cirrhosis	9 (2.2)	8 (1.1)	1 (0.8)	7 (2.3)	4 (4.1)	0.16
ESRD	12 (2.9)	22 (3.0)	2 (1.7)	9 (3.0)	4 (4.1)	0.88
CHF	27 (6.6)	35 (4.8)	4 (3.3)	12 (4.0)	3 (3.1)	0.34
Stroke	13 (3.2)	12 (1.7)	5 (4.2)	7 (2.3)	0 (0.0)	0.13
DM	61 (15.0)	65 (9.0)	13 (10.8)	45 (14.8)	14 (14.4)	0.014
Autoimmune	18 (4.4)	24 (3.3)	6 (5.0)	12 (4.0)	4 (4.1)	0.85
Transplant	10 (2.5)	15 (2.1)	2 (1.7)	6 (2.0)	1 (1.0)	0.92
**NTM treatment,** no (%)	47 (11.5)	112 (15.5)	23 (19.2)	24 (7.9)	5 (5.2)	< 0.001
**All-cause mortality,** no (%)^#^	154 (44.8)	255 (45.2)	56 (59.2)	96 (41.1)	30 (37.8)	0.0031**

*Data are presented as mean ± standard deviation or number (%).*

*^#^The 8-year all-cause mortality rates were estimated from the Kaplan–Meier method.*

*p was from Fisher’s exact test; **p was from the log-rank test; the others were from chi-squared or ANOVA. BMI, body mass index; AFS, acid-fast staining; MABC, Mycobacterium abscessus complex; MAC, Mycobacterium avium complex; MK, Mycobacterium kansasii; RGM, rapid-growing mycobacteria; SGM, slow-growing mycobacteria; NTM, nontuberculous mycobacteria; CKD, chronic kidney disease; COPD, chronic obstructive pulmonary disease; IPF, idiopathic pulmonary fibrosis; ESRD, end-stage renal disease; CHF, heart failure; DM, diabetes mellitus.

### Mortality Analysis of Different Nontuberculous Mycobacteria Species

**FIGURE 3:**
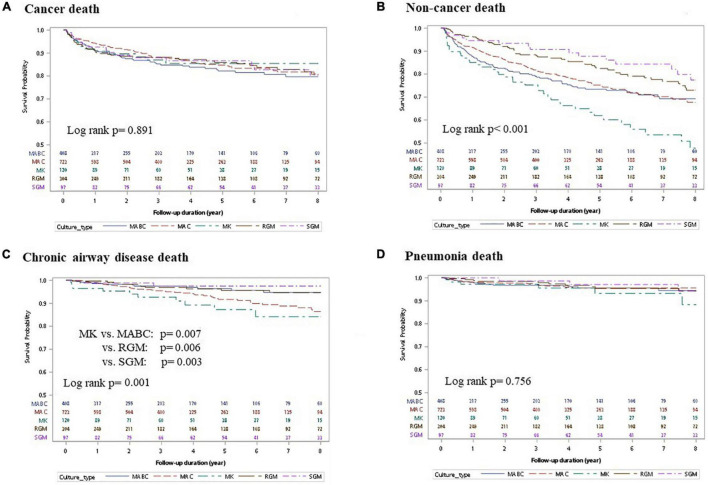
Kaplan–Meier survival curves of nontuberculous mycobacterial lung disease (NTM-LD) according to different NTM species and different death causes. **(A)** Cancer-related death, **(B)** non-cancer-related death, **(C)** chronic airway disease-related death, and **(D)** pneumonia-related death. MABC, *Mycobacterium abscessus* complex; MAC, *Mycobacterium avium* complex; MK, *Mycobacterium kansasii*; SGM, slow-growing mycobacteria.

The highest mortality cause in NTM-LD was cancer, followed by chronic airway disease and pneumonia (Supplementary Table 2). The next three were cardiovascular disease, cerebrovascular disease, and DM, in descending order. Cancer-related mortality was similar among the five subgroups with different NTM species, as determined by KM curves (log-rank *p* = 0.891, Figure 3A). In contrast, non-cancer mortality was significantly different among NTM-LD for the different NTM species (log-rank *p* < 0.001, Figure 3B) and was significantly higher in the MK-LD group than in the other NTM-LD groups (vs. MABC-LD, MAC-LD, RGM-LD, and SGM-LD; pairwise log-rank *p* = 0.012, 0.001, < 0.001 and < 0.001, respectively). The non-cancer mortality rates of MABC-LD and MAC-LD were similar (Supplementary Table 1). Among non-cancer death causes, death caused by chronic airway disease was significantly different among the different NTM-LD groups (Figure 3C). The MK-LD group had significantly higher mortality due to chronic airway disease than those of the MABC-LD, RGM-LD, and SGM-LD groups. The KM survival curve of MAC-LD, which was close to that of MK-LD, also had significantly higher mortality due to chronic airway disease than those of MABC-LD, RGM-LD, and SGM-LD (pairwise log-rank *p* = 0.019, 0.018, and 0.0.12, respectively). However, mortality caused by pneumonia was not significantly different among the NTM groups (Figure 3D).

## Discussion

In this large-scale cohort study, the 8-year mortality in patients with NTM-LD was as high as 45%. NTM species, in addition to old age, male, and comorbidities, were the risk factors for NTM-LD mortality. Notably, the overall 8-year mortality rate was the highest in the MK-LD group, followed by the MAC-LD and MABC-LD groups in that order. Cancer-related NTM-LD mortality rates were similar among the different NTM species, but non-cancer mortality rates were significantly higher in the MK-LD group, followed by the MAC-LD and MAB-LD groups, compared with those of other species. Among the non-cancer mortality, MK-LD and MAC-LD were correlated with death caused by chronic airway disease but not pneumonia.

The results of previous studies investigating the impact of NTM species on mortality are controversial (Kim et al., 2021). Some studies have reported no mortality difference among NTM species, possibly due to small case numbers (Gommans et al., 2015; Aliberti et al., 2020). However, some investigators have suggested that NTM might have species-specific virulence (Stout et al., 2016). Canadian data from 2001 to 2013 indicated that MK-LD had the highest HR of mortality compared to a control population (Marras et al., 2017), but not compared to the NTM-LD mortality of other species. This study echoed the Canadian report and found that MK-LD had the highest rates of all-cause mortality. Furthermore, we reported the impact of NTM species on non-cancer death and mortality due to chronic airway disease.

The various results regarding NTM species on mortality are probably associated with the geographic distribution of NTM species. Generally, MAC is the most common species of NTM-LD, followed by MABC, and both are commonly studied. However, more isolates of *M. xenopi* have been found in Europe than in the United States and East Asia (Bemer et al., 2021). In Taiwan, MK-LD is ranked only as the third most common NTM-LD (Lee et al., 2020). Therefore, analysis of regional epidemiological data and information on the causative species of NTM-LD might be important in the establishment of a prediction model of NTM-LD outcomes. In addition, a thorough discussion of MK-LD and other less common NTM species requires large case numbers. Small cohort studies might have underestimated the between-species differences (Gommans et al., 2015; Aliberti et al., 2020).

*Mycobacterium kansasii* lung disease had the highest all-cause mortality rate, which was mostly contributed by chronic airway disease. MK was highly associated with previous PTB, COPD, and other structural lung diseases (Maliwan and Zvetina, 2005). Notably, MK was reported as a virulent NTM species, and it might cause significant short-term mortality (Huang et al., 2018; Liu et al., 2019). Therefore, it is plausible that MK-LD may have the highest mortality rates for all-cause mortality and chronic airway disease mortality.

Chronic airway disease was found to be the top cause of non-cancer mortality in this study; this finding is similar to that of a study by Lee et al. (2021) in Korea. Yeh et al. (2014) reported that the cumulative proportional incidence of respiratory failure was 10% higher in the NTM population than in the non-NTM population, especially in patients with COPD. The reason could be that NTM-LD is associated with the rapid deterioration of lung function (Huang et al., 2012). MK-LD and MAC-LD may accelerate such a decline more than other NTM-LDs (Lee et al., 2013). COPD with NTM-LD had a higher risk of exacerbation than COPD without NTM isolates (Huang et al., 2012). Another possible reason is that NTM-LD has higher chances of *Pseudomonas aeruginosa* isolates or infections (Hwang et al., 2017). This has been reported as an independent risk factor for COPD exacerbation or mortality (Eklöf et al., 2020). These findings suggest that NTM-LD is a negative prognostic factor of COPD and is associated with advanced COPD. In contrast, this study showed that COPD was the leading comorbidity of MK-LD. Therefore, one possible explanation for the higher chronic airway disease mortality in patients with MK-LD, other than the virulence itself, is that MK infected more patients with advanced COPD.

Cancer-related death was the top first mortality cause of NTM-LD. It is well-known that cancer itself often causes short-term and significant mortality. Some studies on NTM-LD mortality have similarly reported cancer as a risk factor (Jhun et al., 2020). Even so, no difference in cancer-related survival curves was found among different NTM species. In other words, NTM-LD in patients with cancer may be associated with high mortality, regardless of the NTM species.

Regarding pneumonia-related deaths, this study lacked information on pneumonia pathogens. It is difficult to differentiate mortality caused by NTM pneumonia from that caused by other pathogens. However, NTM-LD might be a representative of immune exhaustion (Wang et al., 2021), which results in reduced bacterial clearance and high mortality (Wang et al., 2011). Thus, it is understood that NTM-LD has higher chances of *P. aeruginosa*, *Staphylococcus aureus*, and *Klebsiella pneumoniae* isolates (Hwang et al., 2017). Thereafter, a similar impact on pneumonia-related mortality may exist in NTM-LD by different species.

For radiographical patterns, NTM-LD is mostly categorized into fibrocavitary and nodular bronchiectatic patterns. It is well-known that nodular bronchiectasis has a more indolent course and lower mortality than the fibrocavitary pattern (Moon et al., 2019). Our finding echoed those of other studies that bronchiectasis has protective effects compared with other patterns in NTM-LD, unlike other chronic airway diseases (Marras et al., 2017; Lee et al., 2021).

The strength of this study is the large cohort and long follow-up period for investigating the relationship between NTM species and causes of mortality. However, the study had some limitations. First, the study population was based on one tertiary-care hospital in northern Taiwan. The findings should be validated before application to other regions. Second, this study was conducted retrospectively. The data were not recorded with a standardized protocol. Therefore, some data, such as BMI and smoking, might have been missed. In addition, not every participant received a CT scan of the chest. The incidence of cavitation might have been underestimated, although we assumed that CT scans would be performed if plain films raised any suspicions. Fourth, the data on mortality causes were derived from a government death registry, and the causes beyond the most common ten were unclear. Finally, COPD and NTM-LD had prognostic effects that were additive to each other, especially MK-LD (Huang et al., 2012; Lee et al., 2013). This study had no information about COPD severity. Therefore, further studies might be needed to investigate the relationship of NTM-LD mortality with COPD severity.

## Conclusion

The prognostic impact of NTM species was evident. MK-LD had the highest rates of all-cause, non-cancer, and chronic airway disease mortality. MAC-LD and MABC-LD were the second-highest for non-cancer mortality, whereas MAC-LD was the second-highest for deaths caused by chronic airway disease. The higher NTM species-specific mortality in chronic airway disease might suggest a need for bidirectional screening. In addition, bundle care for chronic airway disease should be added according to NTM species when NTM-LD is diagnosed.

## Data Availability Statement

The raw data supporting the conclusions of this article will be made available by the authors, without undue reservation.

## Ethics Statement

The studies involving human participants were reviewed and approved by the Research Ethics Committees, National Taiwan University Hospital. Written informed consent for participation was not required for this study in accordance with the national legislation and the institutional requirements.

## Author Contributions

C-CS and C-HC were responsible for the conception, design, and critical review. S-MW and C-HC were involved in the data analysis. P-HW, S-WP, and C-CS performed manuscript preparation and writing. All authors contributed to the article and approved the submitted version.

## Conflict of Interest

The authors declare that the research was conducted in the absence of any commercial or financial relationships that could be construed as a potential conflict of interest.

## Publisher’s Note

All claims expressed in this article are solely those of the authors and do not necessarily represent those of their affiliated organizations, or those of the publisher, the editors and the reviewers. Any product that may be evaluated in this article, or claim that may be made by its manufacturer, is not guaranteed or endorsed by the publisher.
