# Supplementary material for: The Impact of Nontuberculous Mycobacteria Species on Mortality in Patients With Nontuberculous Mycobacterial Lung Disease
Source: Front Microbiol. 2022 Jul 7;13:909274. doi: 10.3389/fmicb.2022.909274 (PMC9300315; doi:10.3389/fmicb.2022.909274)
Supplement: Supplementary file 1 [file Data_Sheet_2.docx]

Supplementary Material

**Appendix 1. Microbiological diagnostic criteria of NTM-LD**

We included subjects who fulfilled the microbiological diagnostic criteria of NTM-LD according to the ATS/IDSA diagnosis guideline (Griffith et al.,2007). In brief, the criteria were as follows: (1) two or more isolates of the same species of NTM, (2) fewer than three sets of negative cultures or no other NTM species isolates between two positive NTM cultures, and (3) no diagnosis of pulmonary tuberculosis (PTB) 180 days before or after positive NTM cultures (Winthrop et al., 2011).

**Appendix 2. Details of the NTM species in the RGM and SGM groups.**

The RGM group included *M. chelonae*, *M. fortuitum*, *Mycobacterium vaccae*, and *M. mageritense*. The SGM group included *M. celatum*, *M. gordonae*, *M. phlei*, *M. scrofulaceum*, *M. smegmatis*, *M. szulgai*, and *M. terrae*.

**Appendix 3. Underlying coding**

The comorbidities, including cancer, asthma, COPD, bronchiectasis, pneumoconiosis, IPF, cirrhosis, end-stage renal disease (ESRD), cerebrovascular disease (CVA), CHF, DM, autoimmune diseases (systemic lupus erythema, rheumatoid arthritis, Sjőgren's syndrome, polymyositis, and dermatomyositis), and solid organ transplantations, were defined by the diagnostic coding in medical records.

**Appendix 4. Death cause of cardiovascular disease and cerebrovascular disease**

Mortality caused by coronary artery disease, congestive heart failure (CHF), other kinds of heart diseases, and aortic dissection were classified as cardiovascular diseases. Mortality causes due to stroke and Parkinsonism were categorized as cerebrovascular diseases.

**Reference**

Griffith, D. E., Aksamit, T., Brown-Elliott, B. A., Catanzaro, A., Daley, C., Gordin, F., Holland, S. M., Horsburgh, R., Huitt, G., Iademarco, M. F., Iseman, M., Olivier, K., Ruoss, S., von Reyn, C. F., Wallace, R. J., Jr, Winthrop, K., ATS Mycobacterial Diseases Subcommittee, American Thoracic Society, & Infectious Disease Society of America (2007). An official ATS/IDSA statement: diagnosis, treatment, and prevention of nontuberculous mycobacterial diseases. *Am. J. Respir. Crit. Care Med.* *175*(4), 367–416.

Winthrop, K. L., Baxter, R., Liu, L., McFarland, B., Austin, D., Varley, C., Radcliffe, L., Suhler, E., Choi, D., & Herrinton, L. J. (2011). The reliability of diagnostic coding and laboratory data to identify tuberculosis and nontuberculous mycobacterial disease among rheumatoid arthritis patients using anti-tumor necrosis factor therapy. *Pharmacoepidemiology and drug safety*, *20*(3), 229–235.

**Table S1.** Pairwise log rank test between Kaplan–Meier survival curves of each two subgroups of nontuberculous mycobacterial lung disease (NTM-LD) according to NTM species.

(A). Overall survival curves according to all-cause mortality

| **NTM species** | **NTM species** | **p-value** |
| --- | --- | --- |
| MK | MABC | 0.10 |
| MK | MAC | 0.011 |
| MK | RGM | 0.00058 |
| MK | SGM | 0.0018 |
| MABC | MAC | 0.25 |
| MABC | RGM | 0.024 |
| MABC | SGM | 0.049 |
| MAC | RGM | 0.11 |
| MAC | SGM | 0.10 |
| RGM | SGM | 0.54 |

(B). Survival curves of non-cancer mortality

| **NTM species** | **NTM species** | **p-value** |
| --- | --- | --- |
| MK | MABC | 0.012 |
| MK | MAC | 0.0014 |
| MK | RGM | < 0.0001 |
| MK | SGM | 0.00007 |
| MABC | MAC | 0.51 |
| MABC | RGM | 0.013 |
| MABC | SGM | 0.028 |
| MAC | RGM | 0.025 |
| MAC | SGM | 0.034 |
| RGM | SGM | 0.42 |

(C). Survival curves of chronic airway disease related mortality

| **NTM species** | **NTM species** | **p-value** |  |
| --- | --- | --- | --- |
| MK | MABC | 0.0026 | |
| MK | MAC | 0.18 |  |
| MK | RGM | 0.0035 |  |
| MK | SGM | 0.010 |  |
| MABC | MAC | 0.019 |  |
| MABC | RGM | 0.94 |  |
| MABC | SGM | 0.45 |  |
| MAC | RGM | 0.018 |  |
| MAC | SGM | 0.032 |  |
| RGM | SGM | 0.45 |  |

**Table S2.** Causes of death stratified by species of non-tuberculous mycobacteria

| **Total death**  **(n=591)** | **MABC**  (n=154) | **MAC**  (n=255) | **MK**  (n=56) | **RGM**  (n=96) | **SGM**  (n=30) | **p values** |
| --- | --- | --- | --- | --- | --- | --- |
| Cancer | 60 (39.0) | 94 (36.9) | 14 (25.0) | 44 (45.8) | 15 (50.0) | 0.076 |
| Chronic airway disease | 12 (7.8) | 47 (18.4) | 11 (19.6) | 10 (10.4) | 2 (6.7) | 0.011 |
| Pneumonia | 14 (9.1) | 23 (9.0) | 6 (10.7) | 10 (10.4) | 3 (10.0) | 0.97* |
| Cardiovascular diseases | 7 (4.6) | 12 (4.7) | 2 (3.6) | 9 (9.4) | 0 (0.0) | 0.31* |
| Cerebrovacular diseases | 11 (7.1) | 11 (4.3) | 2 (3.6) | 3 (3.1) | 3 (10.0) | 0.36* |
| DM | 5 (3.3) | 6 (2.4) | 2 (3.6) | 1 (1.0) | 0 (0.0) | 0.75* |
| Others | 45 (29.2) | 62 (24.3) | 19 (33.9) | 19 (19.8) | 7 (23.3) |  |

* p was from Fisher’s exact test; the others were from ANOVA.

Data are presented as number (percentage)

DM: diabetes mellitus

**Figure S1**. Flow chart of subjects’ enrollment.


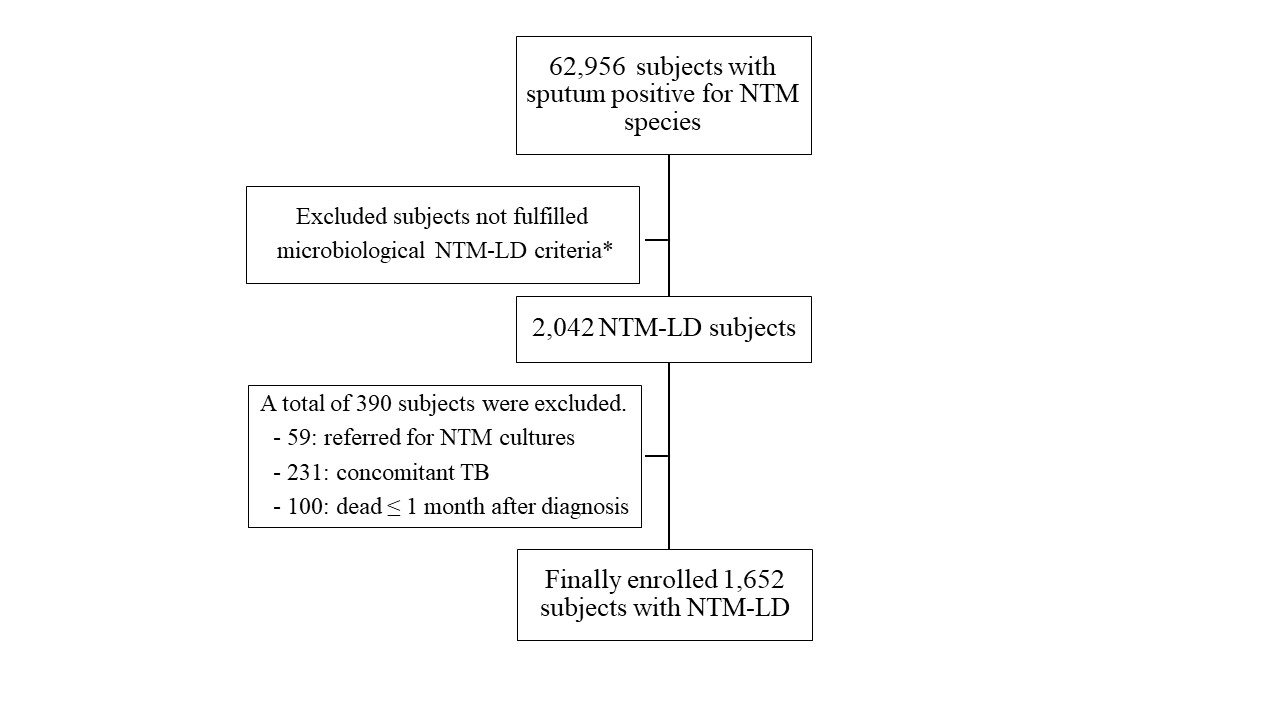


* NTM-LD criteria: The diagnostic criteria were all of these four items: (1) two or more isolates of the same species of NTM within one year, (2) three or more sets of negative cultures between two positive NTM cultures, (3) other NTM species identified between the two positive cultures of a certain NTM species, (4) no clinical or microbiologic diagnosis of pulmonary tuberculosis (PTB) 180 days before or after positive NTM culture.
